# Fatigue Life Improvement of Cracked Aluminum 6061-T6 Plates Repaired by Composite Patches

**DOI:** 10.3390/ma14061421

**Published:** 2021-03-15

**Authors:** Armin Yousefi, Saman Jolaiy, Reza Hedayati, Ahmad Serjouei, Mahdi Bodaghi

**Affiliations:** 1 Department of Engineering, School of Science and Technology, Nottingham Trent University, Nottingham NG11 8NS, UK; yousefi.armin@ut.ac.ir (A.Y.); ahmad.serjouei@ntu.ac.uk (A.S.); 2 School of Mechanical Engineering, College of Engineering, University of Tehran, P.O. Box, Tehran 11155-4563, Iran; samanjolaiy@ut.ac.ir; 3 Department of Aerospace Structures and Materials, Faculty of Aerospace Engineering, Delft University of Technology (TU Delft), Kluyverweg 1, 2629 HS Delft, The Netherlands; rezahedayati@gmail.com

**Keywords:** aluminum 6061-T6, composite patch, finite element modeling, plasticity, fatigue life

## Abstract

Bonded patches are widely used in several industry sectors for repairing damaged plates, cracks in metallic structures, and reinforcement of damaged structures. Composite patches have optimal properties such as high strength-to-weight ratio, easiness in being applied, and high flexibility. Due to recent rapid growth in the aerospace industry, analyses of adhesively bonded patches applicable to repairing cracked structures have become of great significance. In the present study, the fatigue behavior of the aluminum alloy, repaired by a double-sided glass/epoxy composite patch, is studied numerically. More specifically, the effect of applying a double-sided composite patch on the fatigue life improvement of a damaged aluminum 6061-T6 is analyzed. 3D finite element numerical modeling is performed to analyze the fatigue performance of both repaired and unrepaired aluminum plates using the Abaqus package. To determine the fatigue life of the aluminum 6061-T6 plate, first, the hysteresis loop is determined, and afterward, the plastic strain amplitude is calculated. Finally, by using the Coffin-Manson equation, fatigue life is predicted and validated against the available experimental data from the literature. Results reveal that composite patches increase the fatigue life of cracked structures significantly, ranging from 55% to 100% for different applied stresses.

## 1. Introduction

Nowadays, composite patches, due to their unique properties such as lightness, ease of application, high flexibility, and high stiffness, are increasingly being used in industrial applications such as aerospace structures and automobile industries. Investigation of the tensile strength and fatigue behavior of aluminum alloys is still capturing the attention of researchers. Traditional methods of repairing aluminum alloys, for instance, mechanical fastening, could lead to new defects. Composite patches, unlike traditional methods, do not cause any damage to the parent plate and can be replaced several times [1,2,3,4,5,6]. Fiber-reinforced polymer composites are the right choice for being used as a patch since these materials have a high stiffness-to-weight ratio, good fatigue resistance, and excellent corrosion resistance [7,8,9,10].

Using composite patches for repairing structures was first introduced by Baker et al. [11] in the early 1970s in Australia for military aircraft. Afterward, this technology was implemented for repairing structures in the aerospace and automobile industries [12]. Several researchers have analyzed the repairing efficiency of composite patches and have done several optimization analyses on the patch design. Seo et al. [13] investigated the fatigue crack growth in thick aluminum plates repaired by a composite patch. They conducted an experimental study for 10 mm thick specimens repaired by graphite/epoxy patches. The stress intensity factor was determined by both experimental test and finite element method (FEM), and the results were compared. Ergun et al. [14] examined the fatigue life of damaged aluminum 2024-T3 reinforced by composite patches under the hygrothermal effect. The effect of patch thickness and humidity was analyzed. The results revealed that the numbers of patch layers and hygrothermal conditions significantly affect fatigue life.

The FEM is an excellent technique to analyze the effect of different repair process parameters on the overall performance of structures repaired by polymer composite patches [8,9,14,15,16,17,18,19]. Calculating stress intensity factor (SIF) is a reliable approach to describing crack growth behavior [15,17,19,20,21]. Ouinas et al. [15] studied the effect of the patch geometry and adhesive properties on the efficiency of the repair process. They calculated the stress intensity factor using finite element (FE) models. The results indicated that increasing the patch diameter reduces the SIF. Adhesive properties also have a significant effect on the repair output. Toudeshky et al. [16] investigated the residual thermal stresses in structures repaired by a composite patch. Using numerical and experimental approaches, they examined the effect of thermal expansion coefficient mismatch, which leads to residual thermal stresses on the crack-front shape. Results indicated that the thermal residual stresses did not affect the stress intensity factor at the crack-front in the aluminum 2024-T3 plate repaired by patch. Albedah et al. [17] analyzed patch dimensions’ influence on the efficiency of overall patch performance numerically and experimentally for aluminum 2024-T3 and aluminum 7075-T6. 3D FEM was performed to calculate the SIF to evaluate the patch’s effectiveness. Results indicated that increasing the patch length leads to decreasing the fatigue life of repaired plates. Dai et al. [8] studied the effects of resin properties and composite patch configurations on the repair process of the damaged aluminum alloy plates. FEM and experimental studies were carried out to examine the failure of the repaired structures under tensile loadings. Observation revealed that damage was initiated in the layers adjacent to the crack surface. Moreover, delamination and fiber breakage is the main reason for the failure of the composite patch. Mohammadi [18] investigated the effect of different parameters such as the thickness of a cracked plate on the performance and durability of a one-sided composite patch repairing process. Results indicated that the efficiency of the repair process firmly depends upon the parent plate thickness and patch material. Yousefi et al. [19] studied the effect of composite and nanocomposite patch volume fraction on the performance of the repair process. They examined the impact of the patch and adhesive thickness on the efficiency of the repair process. 3D FE analysis was carried out to determine the SIF. Results indicated that SIF significantly depends on the patch stiffness. Hosseini et al. [22] investigated the fatigue behavior of cracked aluminum 1050 repaired by glass/epoxy patches numerically and experimentally for different stress ratios ranging between 0 to 1. Results indicated that by employing the double-sided composite patch, fatigue life is improved significantly for stress ratios 0 and 0.5. Khan Mohammad et al. [23] examined the effect of different shapes of composite patches on the efficiency of the repair process experimentally and numerically. Both numerical and experimental studies showed that composite shape has a significant effect on the repair performance, and rectangular patches provide the most efficient repair compared to the triangular shape. Recent studies show that several parameters could affect the fatigue life improvement, such as the minimization of stress concentration, which improves fatigue life, and environmental condition also may influence the fatigue life [24,25]. Most of the previous research studies have investigated the effect of composite patch on the crack growth or fatigue life by calculating SIF by employing FEM. However, to the authors’s best knowledge, no research work has been carried out on determining the fatigue life of damaged aluminium plates repaired by composite patches employing a strain-based approach. 

In the present study, the effect of applying double-sided glass fiber/epoxy composite on the fatigue life improvement of cracked aluminum 6061-T6 plate is investigated numerically. 3D finite element analysis is performed to investigate the effect of glass fiber/epoxy patch on the fatigue life of damaged structures. In this regard, the steady-state hysteresis loop is determined, from which the plastic strain is calculated. It is worth mentioning that since the strain-based approach is based on the plastic strain, to gain more reliable results and calculate strain, the cylindrical volume (part) as representative volume element is considered near the crack zone in which the strain is calculated by volume averaging of strain from all elements in this specified cylindrical-shaped volume. To the best of the authors’ knowledge, no research work uses this approach (volume averaging) to calculate the strain considering the strain-based approach. Finally, the fatigue life of different configurations (undamaged plate, unrepaired damaged plate, and repaired damaged plate) is predicted. Therefore, the main novelty of this study is employing the strain-based approach to investigate the effect of applying glass fiber/epoxy composite patch on fatigue life in which the strain is calculated using representative volume element (cylindrical-shaped volume).

## 2. Materials and Models

**Figure 1:**
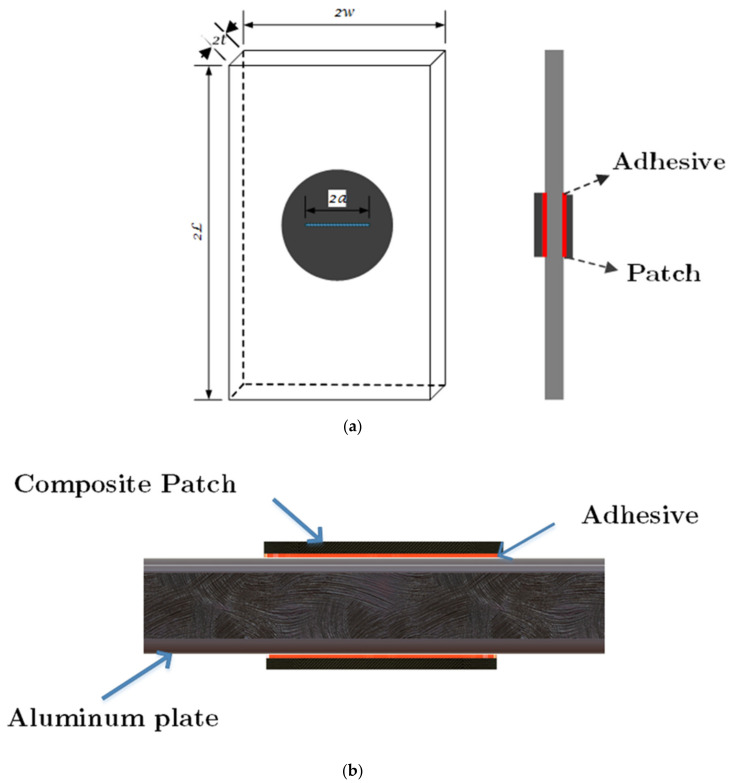
Geometry of repaired damaged aluminum plate: (**a**) Perspective view and (**b**) Front view.

The centrally cracked aluminum 6061-T6 plate (subjected to rolling process) with an initial crack length of a = 2 mm repaired by the composite patches is illustrated in Figure 1. The length (2*L*), width (2*w*), and thickness (2*t*) of the aluminum plate are 500 mm, 125 mm, and 12.5 mm, respectively. As shown in Figure 1a, the crack (blue line) is in the plate’s center and perpendicular to the applied load. The patch is bonded to the cracked plate by means of Araldite 2015 (Huntsman Corporation, The Woodlands, TX, USA) [26]. The initial mechanical properties of the plate and adhesive are listed in Table 1. The Johnson–Cook material model [27] was used to model the elastic-plastic behavior of the aluminum plate. This material model is implemented to model material behavior in large deformations, and it considers the isotropic hardening characteristic. In this study, since the structures undergo large deformation extents, the Johnson–Cook material model is employed. The Johnson–Cook constants for Al6061-T6 (Aluminum Alloys) [28] are listed in Table 2. The mechanical properties of glass fiber/epoxy composite patch are extracted from the work of Devireddy et al. [29], which is reported in Table 3.

### 2.1. Numerical Modeling

**Figure 2:**
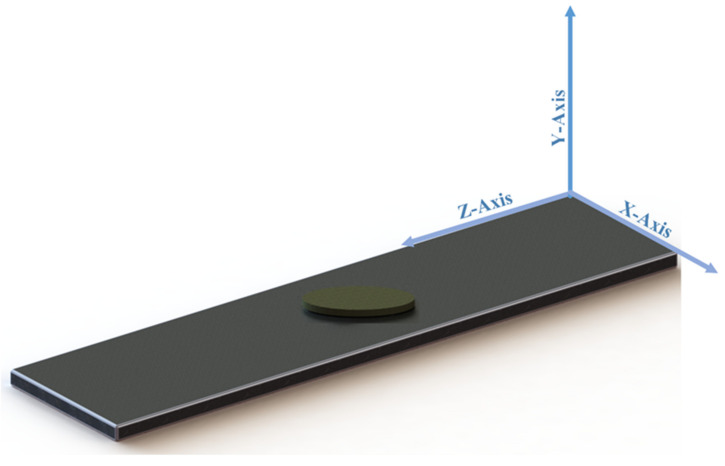
FE model of cracked aluminum plate repaired by composite patches.

**Figure 3:**
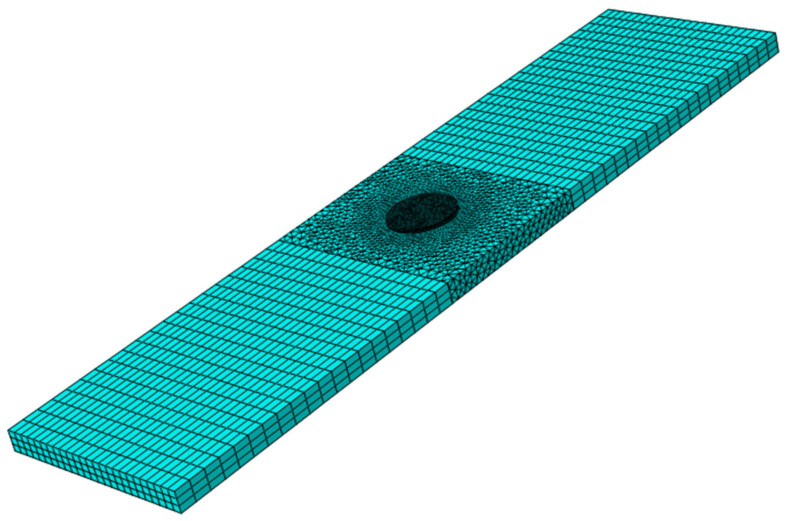
A discretized model of the repaired aluminum plate containing a surface crack.

The cracked aluminum 6061-T6 plate under cyclic loading was analyzed by employing Abaqus software (V. 6.14, Dassault Systems, France). Figure 2 illustrates a typical 3D FE model of a cracked aluminum plate repaired by a composite patch. The FE model consists of three parts: the cracked aluminum plate, the adhesive layer, and the patches. The contact between the patch–adhesive interface and the adhesive–plate interface is assumed to be a perfect bond. The perfect bond is applied using *Tie* constraint in Abaqus software. The perfect bond is used to define surface-to-surface contact interaction in which the rotational and translational motion is equal between two connected surfaces. Figure 3 shows a discretized model of the repaired aluminum plate containing a surface crack. In order to discretize the repaired plate, the plate was sectioned into three parts using the Partition tool in Abaqus software. 3D 8-node linear hexahedral elements of type C3D8R were used to mesh the areas far away from the crack, while 10-node tetrahedral elements of type C3D10 were used to mesh the area in the neighborhood of the crack (cylindrical-shaped volume). To obtain more accurate results, the element size around the cracked region was smaller than the element size of the area far away from the crack. Eight-node linear wedge cohesive elements of type COH3D6 are utilized to mesh the adhesive parts. Also, linear tetrahedral elements of type C3D4 were used to mesh the patch. The boundary conditions applied on the structures that stimulate a relevant fatigue test are reported in Table 4. In this study, to examine the effect of patches on fatigue life improvement at different stress amplitudes, different cyclic displacements were applied.

**Figure 4:**
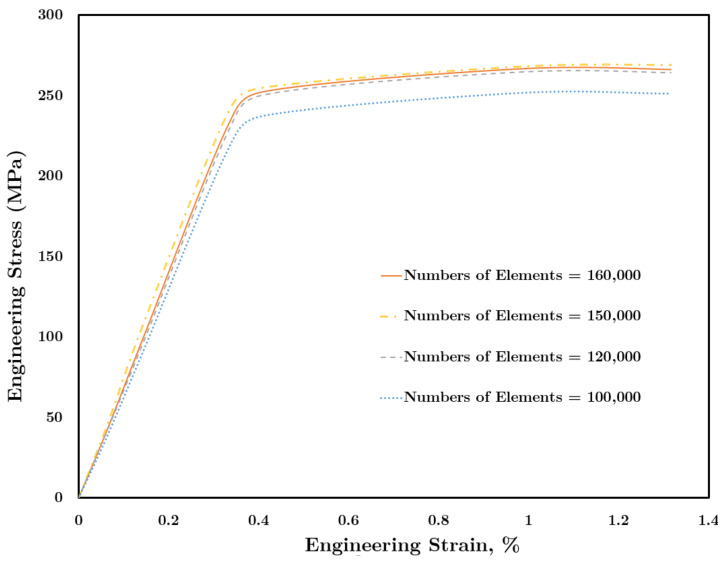
Effect of the number of elements on the engineering stress–strain curve.

In the present study, mesh independence analysis was also performed. For this aim, the element size was reduced (in other words, the number of elements was increased) systematically until the resulting engineering stress–strain curves converged to the same amount. As indicated in Figure 4, the stress–strain curves of the repaired plate converged for element numbers between 120,000 and 160,000. Therefore, in the present study, the element numbers implemented are at least 160,000.

### 2.2. Fatigue Life Analysis

In the present study, in order to determine the fatigue life, the strain-based approach was employed. In this regard, the stress–strain hysteresis loop was plotted. The total strain amplitude was determined, which was divided into two components, elastic strain and plastic strain [31]. By considering the hysteresis loop, the plastic strain can be determined by obtaining the intercept of the loop curve on the strain axis. The stress-life curve could be linearized on the log scale. The curve is described by:

(1) $$\sigma_{a}={\overset{´}{\sigma }}_{f}{\left(2N_{f}\right)}^{b}$$

where $2N_{f}$ is the number of life cycles until failure, ${\overset{´}{\sigma }}_{f}$ is the fatigue strength coefficient, and b is the fatigue strength exponent. Coffin and Manson [31] found the strain-life data could also be linearized on the log scale and be expressed as:

(2) $$\frac{\Delta \varepsilon_{ave-p}}{2}={\overset{´}{\varepsilon }}_{f}{\left(2N_{f}\right)}^{c}$$

where $\Delta \varepsilon_{ave-p}/2$ is the volume averaging plastic strain amplitude, ${\overset{´}{\varepsilon }}_{f}$ is the fatigue ductility coefficient, and *c* is the fatigue ductility exponent.

It is worth mentioning that in order to calculate the strain, the homogenized variables (strain) are determined by volume averaging from all elements in the cylindrical-shaped volume around the crack. This specified volume has a diameter equal to the patch’s diameter; the height equals the plate thickness, and the crack is exactly in the center of this cylinder. The total strain is calculated as:

(3) $$\varepsilon_{ave}=\frac{1}{V_{m}}\int \varepsilon_{m}dV$$

In Equation (3), $\varepsilon_{ave}$ is volume averaging total strain (consist of elastic and plastic strain), ε_m_ is the local strain in each element, and $V_{m}$ is the total volume of the specified cylindrical-shape part. After calculating volume averaging total strain by Equation (3), the steady-state hysteresis loop is plotted for different conditions in Abaqus software, the volume averaging plastic strain amplitude is determined.

In the next step, to predict the fatigue life, Equation (2) was implemented, so the number of cycles to failure was calculated using a MATLAB (MathWorks, Natick, MA, USA) code based on the Newton–Raphson method. The material constants in Equations (1) and (2) are reported in Table 5 [32].

## 3. Results

### 3.1. Validation

**Figure 5:**
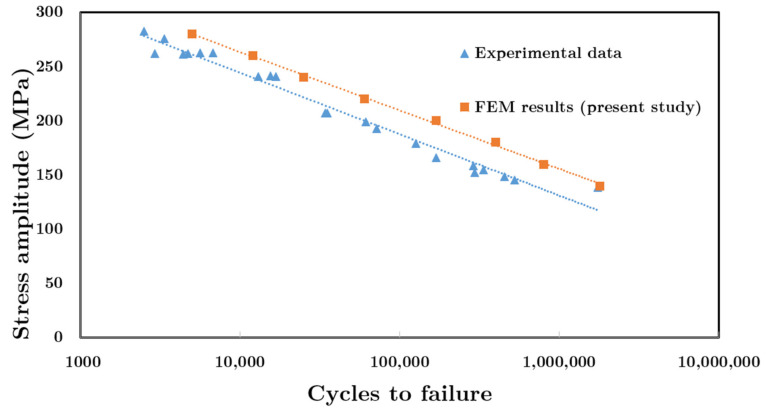
Stress amplitude versus cycles for undamaged aluminum 6061-T6 for both FEM in the present study and experimental data [33].

To verify the proposed model, FEM results were compared with available experimental data [33]. In order to validate the proposed model in the present study, the dimensions of the aluminum plate for comparison of FEM results with experimental data were chosen based on fatigue test standard reported in Ref. [33]. Figure 5 compares the FEM results of the fatigue life for the undamaged aluminum 6061-T6 plate for different stress amplitude against the available experimental data [33]. As shown in Figure 5, there is a good agreement between experimental data and FEM results. The maximum differences between experimental and FEM results are about 20%, although, at a stress amplitude of 140 MPa, this difference is less than 3%. It should be mentioned that at stress amplitude of Δ*σ*/2 =260 MPa, there is about a 23% difference in the numbers of cycles to failure reported by the experimental study. Therefore, these differences between the proposed model and experimental data (ranging from 3% to 20%) could be acceptable for the proposed model in the present study.

### 3.2. Hysteresis Loop

**Figure 6:**
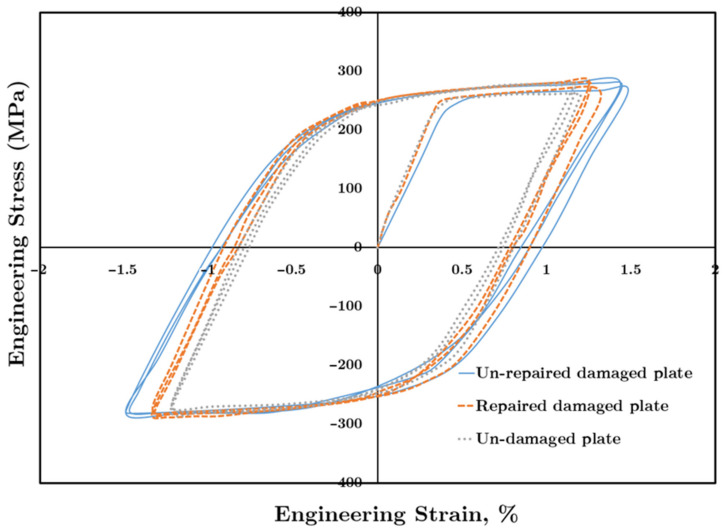
Hysteresis loop for different plate conditions.

Figure 6 shows a typical hysteresis loop for three types of aluminum plates, namely unrepaired damaged aluminum plate, repaired damaged aluminum plate, and the undamaged plate. 

The stress–strain hysteresis loop is drawn in Figure 6 for the fatigue loading condition of stress ratio of R = −1, and stress amplitude of Δ*σ*/2 = 280 MPa is applied to all the cases. At σ=0, the total strain equals plastic strain. According to Equation (2), by increasing the plastic strain amplitude, the fatigue life decreases. Figure 6 shows that the undamaged plate has the lowest strain extent at σ=0, which equals plastic strain amplitude. Furthermore, the unrepaired damaged plate has the highest plastic strain amplitude. The strain extent of the curve of the repaired damaged plate lies in the area between the curves of the undamaged plate and the unrepaired damaged plate, which demonstrates the improvement that repairing the damaged plate has made.

### 3.3. Fatigue Life Prediction

**Figure 7:**
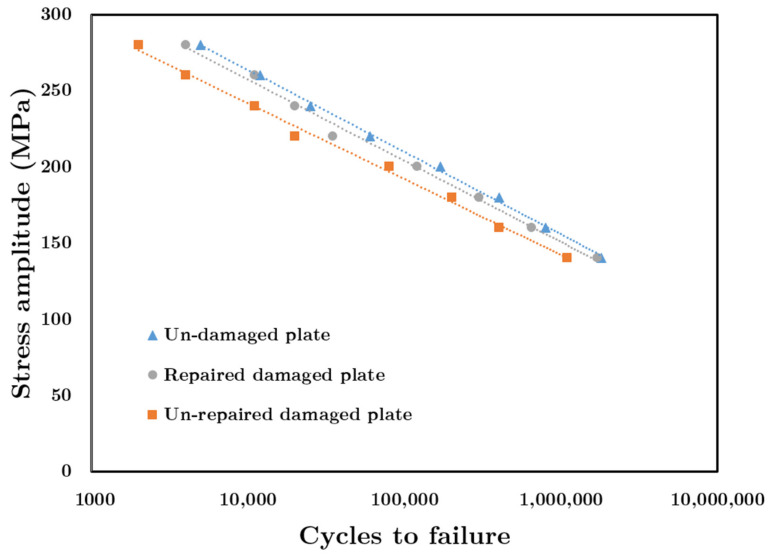
Stress amplitude versus fatigue life for different plate conditions.

As partially mentioned above, in order to predict the fatigue life of both the repaired and unrepaired cracked aluminum plates, first, the hysteresis loop is determined. In this regard, to calculate the hysteresis loop, stress amplitude (y-axis in Figure 7), the amount of stress in the area far from the crack area, is calculated. However, the strain is calculated in the specified cylindrical part. Therefore, at a stress amplitude of 150 MPa (far from crack), the stresses in some elements in the cylindrical part reach yield stress, so plastic strain exists and is determined by Equation (3). Therefore, Equation (2), which is based on plastic strain amount, can be employed even though the stress far from the crack area does not reach the yield amount. The hysteresis loops for three cases (undamaged, damaged, and repaired damaged plate) at each specific stress amplitude are drawn. Afterward, the plastic strain amplitude is calculated based on drawn hysteresis loops for each plate and at each specific stress amplitudes. Finally, by using the Coffin–Manson equation, fatigue life is predicted.

Figure 7 compares the fatigue life of unrepaired and repaired aluminum plates to one another as well as to the fatigue life of the undamaged plate. Figure 7 is a semi-log graph in which the x-axis (cycle to failure) has a logarithmic scale. As shown in this figure, using a composite patch at high-stress amplitude is more effective on the increasing fatigue life as compared to when it is used in low-stress amplitude. At Δσ/2 =280 MPa, applying a double-sided composite patch on the damaged aluminum plate increases the logarithmic fatigue life by almost 100%. In comparison, at Δσ/2 =140 MPa, applying a double-sided composite patch increases the logarithmic fatigue life by 55%.

The main reason for the improvement of fatigue life is that by applying the composite patch at each applied stress amplitude (which is determined far away from the crack area), the stress in the area near the crack is decreased and is lower than the plate without the composite patch. Therefore, the amount of plastic strain is decreased, so according to Equation (2), the numbers of cycles to failure ($N_{f})$ increases. In the higher applied stresses amplitude (for example, at 280 MPa), applying a composite patch could even have a more significant effect on reducing stress (as well as plastic strain) in the area near the crack in comparison to lower applied stresses (at 140 MPa). Therefore, at higher applied stress, the applied composite patch is more effective.

## 4. Conclusions

In the present study, the effects of applying a composite patch on the fatigue life of cracked aluminum plates were studied numerically. In this regard, the stress–strain hysteresis loops were drawn for three specimens: cracked aluminum plate, repaired aluminum plate, and undamaged aluminum plate. Based on the strain-based approach, the fatigue life was calculated for three types of specimens. The results showed that:

1. The aluminum plate repaired by composite patches has a narrower hysteresis loop in the strain direction compared to the unrepaired damaged plate. Therefore, the plastic strain amplitude of the repaired plate is lower than the unrepaired plate. For example, at Δσ/2 =280 MPa, the plastic strain amplitude of the repaired plate becomes 50.3% less than the plastic strain amplitude of the unrepaired damaged plate.
2. Using the composite patch improves the logarithmic fatigue life significantly (at Δσ/2 =280 MPa, by 100% and at  Δσ/2 =140, by 55%).
3. The higher the applied load level, the higher the effect of applying a double-sided composite patch is on the damaged aluminum plate’s fatigue life improvement.

In this paper, the modeled structure was composed of materials that are common materials used in industry. For instance, Araldite 2015 (Huntsman Corporation, The Woodlands, TX, USA) as an adhesive was employed and used for applying patches to the aluminum plate; in addition, glass fiber/epoxy composite was used as a patch, which is highly utilized in industry for repairing damaged structures. Furthermore, the results in the present study were compared with available experimental data to verify the proposed model. Therefore, the results reported in this article could be reliable and give new insight into the fatigue life improvement of the repaired aluminum plate by composite patches.

## Figures and Tables

**Table 1:** Mechanical properties of aluminum plate and adhesive [26,30].

Materials	Materials Behavior	Elastic Modulus (MPa)	Poisson’s Ratio	Tensile Strength (MPa)
**Araldite 2015**	elastic isotropic	1850	0.33	21.63
**Aluminum 6061-T6**	elastic-plastic	72,000	0.35	311

**Table 2:** The Johnson–Cook model constants for Al6061-T6 [28].

A (MPa)	B (MPa)	C_0_	n	m_0_
250	79.7	0.0249	0.499	1.499

**Table 3:** Mechanical properties of glass fiber/epoxy composite patch [29].

	Fiber Volume Fraction (%)	E_1_ (GPa)	E_2_ (GPa)	E_3_ (GPa)	V_12_	V_13_	V_23_	G_12_ (GPa)	G_13_ (GPa)	G_23_ (GPa)
**Glass Fiber /Epoxy**	60	45	12	12	0.28	0.28	0.4	5	5	5.6

**Table 4:** The boundary conditions applied to the structure.

Plane	Z = 0	Y = 0	X = 0	Z = 2L
Boundary Condition	Uz = 0	Uy = 0	Ux = 0	Uz = cyclic displacement

**Table 5:** Materials parameters for aluminum 6061-T6 [32].

Materials Constant	Aluminum 6061-T6
Fatigue Ductility Coefficient,$\;{\overset{´}{\varepsilon }}_{f}$ (mm/mm)	0.77
Fatigue Ductility Exponent, *c*	−1.01
Fatigue Strength Coefficient,$\;{\overset{´}{\sigma }}_{f}$ (MPa)	386
Fatigue Strength Exponent, *b*	−0.036

## Data Availability

Data is contained within the article.
